# Implications of asymptomatic infection for the natural history of selected parasitic tropical diseases

**DOI:** 10.1007/s00281-020-00796-y

**Published:** 2020-03-18

**Authors:** Jorge Alvar, Fabiana Alves, Bruno Bucheton, Louise Burrows, Philippe Büscher, Eugenia Carrillo, Ingrid Felger, Marc P. Hübner, Javier Moreno, Maria-Jesus Pinazo, Isabela Ribeiro, Sergio Sosa-Estani, Sabine Specht, Antoine Tarral, Nathalie Strub Wourgaft, Graeme Bilbe

**Affiliations:** 1 Drugs for Neglected Diseases initiative, Geneva, Switzerland; 2 Institut de Recherche pour le Développement, Université de Montpellier, Montpellier, France; 3 Institute of Tropical Medicine, Antwerp, Belgium; 4 WHO Collaborating Cenre for Leishmaniasis, Instituto de Sakud Carlos III, Madrid, Spain; 5 Swiss Tropical and Public Health Institute, Basel, Switzerland; 6 Institute for Medical Microbiology, Immunology and Parasitology, University Hospital Bonn, Bonn, Germany; 7 ISGlobal, Barcelona, Spain; 8 Drugs for Neglected Diseases initiative, Centro de Investigación de Epidemiología y Salud Pública (CIESP-IECS), CONICET, Buenos Aires, Argentina

**Keywords:** Asymptomatic, Chagas disease, Leishmaniasis, Filariasis, Human African trypanosomiasis, Malaria

## Abstract

Progress has been made in the control or elimination of tropical diseases, with a significant reduction of incidence. However, there is a risk of re-emergence if the factors fueling transmission are not dealt with. Although it is essential to understand these underlying factors for each disease, asymptomatic carriers are a common element that may promote resurgence; their impact in terms of proportion in the population and role in transmission needs to be determined. In this paper, we review the current evidence on whether or not to treat asymptomatic carriers given the relevance of their role in the transmission of a specific disease, the efficacy and toxicity of existing drugs, the Public Health interest, and the benefit at an individual level, for example, in Chagas disease, to prevent irreversible organ damage. In the absence of other control tools such as vaccines, there is a need for safer drugs with good risk/benefit profiles in order to change the paradigm so that it addresses the complete infectious process beyond manifest disease to include treatment of non-symptomatic infected persons.

## Introduction

The term ‘symbiosis’ sensu *lato* encompasses an extraordinary range of relationships between living organisms determined by co-evolution, ranging from being beneficial for both (‘mutualism’) to situations where one kills the other (‘predation’). Within this range of possibilities is ‘parasitism’ (from Greek, ‘para’ = next to, ‘sitos’ = to seat^1^). Parasitism is a long-term relationship between host and parasite that lasts until the symbiosis reaches an imbalance, eventually resulting in host or parasite death. In addition, the sooner a parasite kills the host, the worse the outcome for the parasite’s life cycle ‘goal’; thus, it needs mechanisms to circumvent this limitation. To enable the parasite to survive in the host the parasite requires three things: nutrition, shelter from a hostile environment, and time to reach reproductive maturity and/or to allow vectors an opportunity for transmission, in other words, to ensure that the parasites are perpetuated. Once the parasite ‘loses’ its host, parasite survival becomes challenging, although there are various mechanisms to overcome this. At least two key questions emerge from this kind of relationship: First, how the shared natural history of the parasite and the host ensures a long relationship, and second, why parasitism does not necessarily end in disease.

**Fig. 1:**
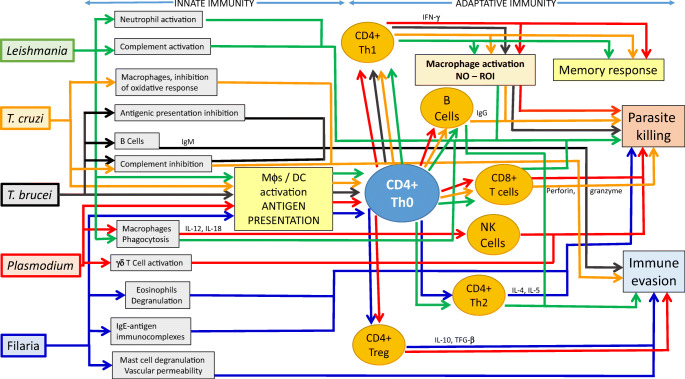
Comparison of the different immune responses to the parasites mentioned in this article

In parasitic diseases, survival strategies developed by helminths are different to those evolved by protozoa, although both follow a basic pattern. Figure 1 summarizes and compares the routes used by the parasites mentioned in this article to evade the immune response and the ways the host is able to eliminate them. All these parasites can avoid the immune response by varying their antigens (not shown in the figure), and they are able to evade innate immune response by following different strategies. The host is able to eliminate parasites through an adaptive immune response that involves antigenic presentation by DCs and macrophages, and the selection and activation of specific T and B cells. Although this route is common for all these parasites, the effector mechanisms against protozoa involve the development of a specific Th1 response, which induces the microbicide activity of macrophages and the cytotoxic activity of CD8+ T cells. In the case of filaria, the protective response is associated with the production of different isotypes of specific antibodies, depending on the development of an effective type Th2 immune response.

Infection is the first contact between the parasite and its host; the host may kill the parasite due to innate and/or acquired immunity, or the parasite may survive due to an efficient mechanism that evades the host response. If the parasite survives, an intriguing dynamic relationship between host and parasite may result; when in balance, the host becomes an ‘asymptomatic carrier’, and when out of balance, the result is disease. Disease manifestation is preceded by a variable period of time known as a pre-patent or incubation period, corresponding to the time required by the parasite to subvert host immunity. Due to co-evolution, it is very common in some parasitic diseases for the number of patients to be low compared with the large number of people with asymptomatic infection (note: in general, an infected person who is asymptomatic is not necessarily a patient).

**Fig. 2:**
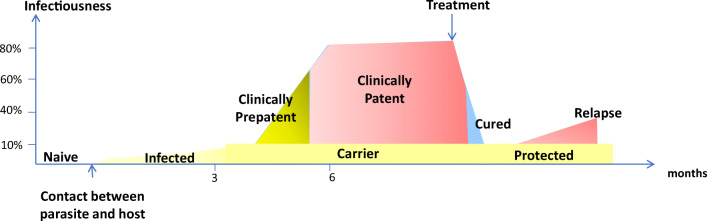
Typical evolution of the infection/disease process resulting in an asymptomatic condition (yellow) or clinically diagnosable disease (red)

Figure 2 summarizes the process of infection, resulting in an asymptomatic condition or clinically diagnosable disease.

### Parasite mechanisms to avoid host immune response

Parasites have developed different mechanisms to manipulate host immunity to ensure appropriate conditions for multiplication in the chronic infection stage. Cellular recognition, activation, and regulation of innate and adaptive host immune responses are altered to allow parasite persistence in host tissues for, at the very least, long enough to multiply and reach the numbers necessary for successful transmission. In fact, many vector borne parasites establish long-lasting chronic infections in order to maximize the probability of transmission [1].

It is well recognized that helminths induce host immune responses that are very different to those induced by protozoa, and, therefore, their evasion mechanisms are different. Intracellular infection is a common mechanism in protozoa that prevents activation of the complement system, together with subverting the T cell response and inhibiting signalling pathways such as NF-kB or the expression of MHC molecules. In helminths, frequently, the release of immunomodulatory molecules, e.g. antiinflammatory or host immunosuppressive cytokines homologues, directly suppresses the host immune response. One strategy shared by protozoa and helminths to evade the host immune response is antigenic variation [2].

### Parasites studied in this review

This review focusses on five insect-borne tropical diseases: Chagas disease, human African trypanosomiasis (HAT), leishmaniasis, filariasis, and malaria, all caused by parasites with different survival mechanisms in the host, leading to very different consequences for epidemiology, and with heterogeneous needs in terms of chemotherapy. Insights into the role of asymptomatic patients arose from a 1-day conference conducted by DND*i* in Geneva on 19 February 2019, entitled ‘Asymptomatic infections, implications for drug development’, aiming to better understand the needs for drug discovery and development, and how to implement products in the DND*i* portfolio. DND*i* has targeted neglected tropical diseases (NTDs) and malaria by making therapies available through registration in endemic countries and/or developing better patient-adapted treatments through improvement in formulations or drug combinations. Over the longer term, DND*i* has defined and refined target product profiles to guide de novo design of tailor-made, patient-adapted, oral therapies, aiming for short treatment duration and addressing all needs in disease populations. A number of low-cost, relatively short-duration therapies have been made available through the work of DND*i* and partners. These include fixed dose combinations of artesunate + mefloquine (ASMQ) and artesunate + amodiaquine (ASAQ) for both adult and paediatric populations with malaria; a combination treatment of nifurtimox eflornithine therapy (NECT) for use in hospital settings, and oral treatments fexinidazole and acoziborole (a potential one-dose, oral drug with a good safety profile currently in advanced phase 3 clinical trials) for treatment of HAT; therapies for Indian, African, and South American leishmaniasis, and other clinical manifestations of leishmaniasis, such as post-kala-azar dermal leishmaniasis and severe forms of leishmaniasis with HIV coinfection; and a paediatric formulation of benznidazole for cases of congenital transmission of Chagas disease. Some of these treatments may be applicable for the treatment of asymptomatic individuals infected with parasites. In addition, innovative oral therapies are in development to meet the duration, efficacy, and tolerability gaps of currently available treatments and to treat human reservoirs of disease (an overview of the DND*i* portfolio and approaches to new therapies is available at www.DNDi.org). In line with DND*i*’s strategy to address patient needs and potential reservoirs of new disease or outbreaks in order to control or eliminate NTDs, this review addresses the potential challenges to disease elimination from individuals with asymptomatic infections who, although not presenting clinical symptoms, may act as reservoirs for infection and foci for new disease outbreaks. The characteristics of asymptomatic infections and their potential for disease generation are discussed below and will be examined in the context of the challenges for disease elimination or control.

#### Chagas disease

More than six million people are estimated to be infected with the causative agent of Chagas disease, *Trypanosoma cruzi* (*T. cruzi*) [3]. Chagas disease presents in two phases, the main clinical characteristic being the lack of symptoms for the acute phase and, if it appears, non-specific symptoms for the chronic phase [4]. Around 95% of those in the acute phase are asymptomatic carriers. This is clinically relevant, because without diagnosis and treatment, people in the acute phase enter into a chronic phase, after which, in the absence of treatment, about 70% will have parasites circulating in their blood and tissues for the rest of their lives without evidence of organ involvement [4].

Host-parasite interactions that lead to such a broad clinical spectrum are still not well understood 110 years after the discovery of Chagas disease. The presence and polymorphism of *T. cruzi* has been considered to be key to this, but up to now, correlations between the pathogenicity, tissue-tropism, or drug-susceptibility [5] of specific *T. cruzi* strains or distinct typing units have yet to be confirmed [6].

Approximately five million people globally are asymptomatic *T. cruzi* carriers, which is highly significant in terms of individual and public health. From an individual health management point of view, people with asymptomatic infection (in the ‘indeterminate clinical form’) are those in which there is evidence of *T. cruzi* infection, but no evidence of organ damage (mainly cardiological or digestive), assessed by (1) non-specific symptoms and (2) low sensitivity tests to detect early organ damage, such as electrocardiogram, chest X-ray, and barium swallow and enema. Even without evidence of organ damage, it is possible that a silent progressive physiopathogenic process has begun, due to the presence of parasites circulating in the blood and those infected tissues where replication takes place [7]. The presence of parasites in blood or tissues can induce damage directly or by inducing microvascular alterations and/or a specific immune response that produces an imbalance in favour of the development of pathogenesis [8].

Lack of evidence of damage does not mean a lack of progression of organ damage, but rather a lack of accurate tools to detect the damage early, delaying diagnosis, and therapeutic options for patients. However, with the diagnostic tools currently available, it is not possible to predict which people with an asymptomatic infection will develop Chagas disease in the future.

Asymptomatic people may contribute to transmission in endemic countries primarily not only through vector transmission but also through mother to child transmission, transfusion of uncontrolled blood and blood products, and organ transplant. Thus, early diagnosis and treatment of asymptomatic carriers of *T. cruzi* is an individual and public health measure that contributes to disease control. The higher the burden of disease in a population in terms of parasitaemia (‘populational parasitaemia’), the greater the need for treatment. Control measures adapted to local scenarios are essential for reducing the number of new cases.

There is currently enough evidence to recommend treatment in people under 18 years old, and women of reproductive age [9], and there is increasing evidence of the value of treating infected people and people with mild chronic disease (in all cases without contraindication) [10–12].

There are limitations to scaling up treatment, such as health workers’ reluctance to prescribe treatment due to the safety profile, and the lack of clear evidence about the clinical benefit of treating asymptomatic adults. Accepted drugs to treat *T. cruzi* infection (benznidazole and nifurtimox) eliminate or reduce parasitaemia but have a suspension rate of around 20% due to poorly tolerated side effects [13, 14].

Inadequate production, and consequently distribution, increasing the barriers to easy and continuous access to treatment [15, 16], is in part due to a random forecast of drug needs.

Nevertheless, in public health terms, diagnosis and treatment of asymptomatic people is a method of primary prevention for congenital and vectorial transmission and of secondary prevention in terms of morbimortality. In addition, administering a drug that tackles chronic infectious Chagas disease has positive effects in terms of well-being, a factor which has not been widely measured up to now; psychological factors and perceptions, even if they are described in this population, have not been studied in terms of the impact on quality of life.

The first research need emerging from the discussion above is better diagnosis and classification of people with *T. cruzi* infection. To do this, more accurate tests to determine organ damage early should be available in countries with a high burden of Chagas disease [17]. Discovery and development of molecular biomarkers of early cardiological and digestive damage, as well as prognostic biomarkers, would be useful tools to address this need [18, 19].

To control transmission dynamics due to asymptomatic infections, and to contribute to reducing their numbers in traditionally and newly endemic areas, more accurate knowledge of the epidemiological situation in each country is needed. Seroepidemiological studies that support more efficient local surveillance systems are necessary to better establish needs and prioritize control measures. Updated local guidelines for case management of individuals infected with *T. cruzi* that are adapted to each context are also necessary [16], and harmonization between currently existing guidelines would facilitate more comprehensive healthcare.

In terms of research, even though in the last decade new drugs were tested in the hope of increasing treatment options for patients with *T. cruzi* infection [11, 12], there remains an urgent need to develop new drugs and new regimens and/or combinations of existing drugs. Drugs and/or drug combinations with good efficacy and, importantly, a better safety profile may increase treatment access for people with asymptomatic *T. cruzi* infection, facilitating better case management at the individual level, and modifying transmission dynamics by reducing population parasitemia. In order to complete cost-effectiveness studies, there is a need for more data, as the clinical impact and quality of life data currently available for asymptomatic people who have received diagnosis and treatment is inadequate.

These needs for Chagas disease were summarized and presented to regulatory regional and global health institutions after agreement between researchers, physicians (at individual and institutional level) and communities in the form of the Santa Cruz letter [20]. Three out of the four measures proposed are in line with the perspectives highlighted in this manuscript.

The first is to expand access to diagnosis and treatment of the disease within the framework of health systems. For asymptomatic people, this means increasing coverage to two thirds of people with *T. cruzi* infection; as a result of implementation, there will be a reduction in the number of new cases and, consequently, a reduction in the burden of the disease, contributing to disease control.

The second is to increase investment in research and development, in alignment with the Sustainable Development Goals, to obtain new, safer, and more effective therapeutic tools. This measure supports not only the strategy to discover and produce new drugs but also the need to obtain new diagnosis and prognosis tools that better define individual risk of developing organ damage.

The third, to improve the surveillance of Chagas disease by establishing compulsory reporting of chronic cases and their complications in the general population, addressed the need for a better surveillance system to support the design of more accurate public health strategies to control *T. cruzi* in the group with the highest burden of infection.

#### Human African trypanosomiasis

HAT is caused by two species of the kinetoplastid protozoan parasite *Trypanosoma brucei*: *T. b. gambiense* (West and Central Africa) and *T. b. rhodesiense* (East Africa) and is transmitted to humans by tsetse flies. Asymptomatic infections may exist for both forms of HAT but latent infections lasting for decades are only documented for *Trypanosoma brucei gambiense* (*T. b. gambiense*) [21, 22]. In contrast to animal trypanosomiasis, investigations into the underlying mechanisms of trypanotolerance in HAT are rare. Few studies have investigated the immune response in individuals with detectable antitrypanosome antibodies but without detectable parasites in blood or lymph node aspirate. In these individuals, elevated levels of the immunosuppressive molecules IL-10 and soluble HLA-G were associated with progression to disease and parasitological confirmation in the months following initial serodiagnosis [23, 24]. In contrast, high levels of inflammatory cytokines were associated with prolonged seropositivity without parasitological confirmation (IL6) or even with probable self-cure (IL8) [23]. Further evidence that immunosuppression due to *T. b. gambiense* infection is a hallmark of disease progression comes from two African patients living in Europe, who, after immunosuppressive therapy, developed neurological signs and eventually were recognized as HAT cases long after they had left Africa [22, 25]. Genotyping parasites from latent and clinical cases failed to show different microsatellite profiles, suggesting that the host rather than the parasite is responsible for the diversity of infection outcomes [26]. APOL1 is a critical molecule in the interaction between trypanosomes and humans. Two kidney disease risk coding variants of the APOL1 gene were associated with differential susceptibility to HAT: the G2 allele was associated with protection against *T. b. rhodesiense* in a Ugandan population, whereas in Guinea, the frequency of the G1 allele was increased in individuals with asymptomatic infections [27, 28]. Previous attempts to identify human genetic factors of resistance/susceptibility to HAT have focused on candidate gene approaches. Recently, a panel of 96 SNPs was tested in several populations from endemic countries as part of the TrypanoGEN project (www.trypanogen.net) [29–34]. Although significant genetic associations were observed at the *APOL1*, *IL6*, *HLAG*, *IL1A*, and *HP* loci, these associations were rather country-specific. This suggests that resistance/susceptibility to HAT is heterogeneous with different genes implicated in different populations depending on history of exposure not only to a variety of pathogens but also to the genetic diversity of trypanosomes across Africa. An example is that the *APOL1* G2 association was not replicated in a different population from Uganda where *rhodesiense* HAT is known to be more ‘chronic’ [29]. Genome-wide association analysis is ongoing to identify new genes with important effects that are common across Africa. Interestingly, recent studies have shown that *T. b. brucei* and *T. b. gambiense* can reside in the skin of infected mice without detectable parasites in the blood and that these skin-dwelling parasites can infect tsetse flies [35]. Preliminary results indicate that dermal trypanosomes are commonly observed in skin biopsies not only from HAT patients but also from non-parasitologically confirmed seropositives (manuscript in preparation). The role of these skin-dwelling trypanosomes in the life cycle within the human host, as well as their infectiousness to tsetse flies, still needs to be elucidated.

In addition to a putative wild or domestic animal reservoir, any undiagnosed human case of *T. b. gambiense* infection may contribute to the sustained transmission of the parasite and therefore jeopardize efforts to eliminate HAT [36]. Reasons for not being diagnosed are diverse, e.g. not showing up during an active screening campaign or having no means to reach a fixed health centre with diagnostic facilities. Also, due to imperfect diagnostic tests, clinically and/or serologically suspect HAT cases may remain unconfirmed by parasitological examination tests, resulting in them not being treated and cured.

A number of unconfirmed seropositive people were followed up for several years and did not develop the disease [21]. These ‘trypanotolerant’ seropositive and asymptomatic cases are often thought to sustain transmission of the parasites in a given focus. However, evidence that these human asymptomatic cases effectively transmit the parasite to the vector is based on very few experiments conducted decades ago [37]. In the absence of recent accurate data on the fraction of human *T. b. gambiense* infections that can be considered asymptomatic and on their infectiousness to the vector, it remains impossible to correctly assess their contribution to transmission. Nevertheless, a study conducted in Zaïre (now D.R. Congo) provides evidence that treatment of parasitologically non-confirmed seropositives (not necessarily asymptomatic) can drastically reduce the annual incidence of HAT [38]. Treatment of seropositive and asymptomatic infections can also be beneficial to the individual person, as illustrated by a report on the treatment of 26 parasitologically non-confirmed seropositive people who became seronegative over time, which was interpreted as evidence of infection before and cure after treatment [39]. Treatment of non-confirmed seropositive people has been proposed on several occasions but has not been widely applied due to the toxicity of the then available drugs. Today, the situation remains unchanged. Pentamidine is still the recommended drug to treat *T. b. gambiense* patients in first stage of the disease, when clinical symptoms are mild or even absent. Moreover, prognostic markers for disease progression are still under investigation [24].

In view of the goals to eliminate and prevent the re-emergence of HAT, understanding the biological mechanisms and epidemiological role of asymptomatic *T. b. gambiense* infections that remain undiagnosed or untreated is critically important. Yet, many questions remain unanswered and deserve further investigation. For example: How frequent are asymptomatic infections and what is their average duration? How infective are they to the vector? What factors may trigger evolution of asymptomatic infections into disease? Will the new drugs under development be able to cure asymptomatic infections? In order to answer these questions and to adapt mathematical transmission models accordingly, it is necessary to invest in improved diagnostics and investigate prognostic markers for disease progression and treatment outcome.

#### Leishmaniasis

Leishmaniasis comprises a group of diseases, all caused by parasites of the genus *Leishmania* and transmitted by sandflies. *Leishmania* is a digenic protozoa that survives in hostile environments, such as the flagellated ‘promastigote’ form in the midgut of the insect vector, and the ‘amastigote’ form in the phagolysosome of the mammalian macrophage. Following infection by the sandfly vector, the parasite multiplies and circumvents the effect of the oxygen cascade and the lytic action of phagolysosomes and the low pH inside the phagolysosome. In cutaneous leishmaniasis, the Th1 response activates such defence mechanisms and the parasite is controlled. In visceral leishmaniasis, the Th2 response is triggered and the amastigotes multiply until no more can be hosted in the vacuole, at which point the macrophage bursts and the parasites released invade other macrophages of the reticuloendothelial system and progressively provoke enlargement of the spleen and liver, the main symptom of visceral leishmaniasis. The macrophage is a key immune cell persistently infected by the parasite, which over one-third of macrophage genes are activated in the presence of parasite illustrates the complexity of this relationship [40].

Various factors determine the severity of leishmaniasis, ranging from asymptomatic to fatal, such as the *Leishmania* species, parasite virulence, and immune response of the infected individual. Although ‘virulence’ can be a simplistic approach, a didactic model stresses the role of two molecular determinants for pathogenicity in natural *Leishmania* infection, the invasive/evasive determinants allowing the invasion of the macrophage by the parasite and its survival, and molecules called pathoantigens involved in tissue damage [41]. For cases of HIV-*Leishmania* co-infection or during other interventions, such as chemotherapy, additional parameters should be considered.

Leishmaniasis is a poverty-related disease, with a great diversity of clinical presentations ranging from self-healing cutaneous forms to the more severe visceral form (VL), which is fatal if not treated. Although the global incidence of VL has decreased in South Asia in recent years due to elimination efforts [42], it remains unchanged with risk of epidemics in Eastern Africa, and in other endemic areas such as Latin America and Europe, the incidence of VL is growing and its distribution is expanding because of climate change, environmental transformation, and migration.

VL is caused by *L. donovani* and *L. infantum* parasites. As mentioned, after infection, progression to clinical VL depends on the balance between multiple factors that promote or prevent the multiplication and expansion of parasites in the body: virulence, the microbiome, factors transmitted through the bite, nutritional status of the host and social conditions, age, immunosuppression, concomitant diseases, etc. The complexity of the response makes it difficult to predict the outcome of the infection, but it is known that most people infected with *Leishmania* remain asymptomatic. In VL endemic areas, the ratio of asymptomatic versus active VL cases is variable: 2.4:1 in Sudan, 4:1 in Kenya, 5.6:1 in Ethiopia, between 4:1 and 17:1 in the Indian subcontinent, and 50:1 in Spain [43]. These values reflect not only differences in the virulence of the strain involved and the characteristics of the host but also differences in the design of the studies and the methods used to identify asymptomatic infections. In addition, levels of parasite transmission fluctuate greatly within an endemic area, with a gradient of prevalence for asymptomatic *L. infantum* infection ranging from 8–12% to 35–38% [44, 45].

The various studies to determine the proportion of asymptomatic individuals who progress to VL report different figures. In India, it has been described that 2 to 23% of asymptomatic individuals developed symptoms within 1 year [46, 47], and this progression to VL was strongly associated with a positive molecular test (quantitative PCR) in blood, or having a high value for a serological test (direct agglutination test: DAT, or rK39-ELISA) [48]. On the contrary, a positive cellular test means a very low risk of subsequent VL [48, 49]. In a recent cross-sectional survey done in a post-outbreak area in Spain, 164 out of 804 (20.7%) individuals were identified as asymptomatic by using a cellular test (whole blood stimulation assay, WBA) and none of these subjects developed visceral leishmaniasis after 4 years of follow-up [45].

The term asymptomatic *Leishmania* infection was used for the first time in 1974 by Pampiglione; four decades later, the definition is still unclear. An asymptomatic person is usually regarded as someone from an endemic area who shows an immune response (either antibodies or a specific cellular response) against *Leishmania*, or who has parasites—or parasite DNA—in the blood, but remains healthy.

There is no single universally accepted assay to identify asymptomatic infection. Disadvantages of using serological tests include unsatisfactory results when parasite transmission is low or intermittent [50] and the fact that serological markers can revert to negative within 4 months [51]. Cell immunity induced by *Leishmania* infection usually remains positive for several years, sometimes even throughout an individual’s life. Thus, seropositivity rates in population-based studies are considerably lower than cellular reactivity rates [52]. The leishmanin skin test (LST) has been widely used to identify asymptomatic individuals [44, 49] but cannot currently be used in a number of countries since no GMP leishmanin reagent has been produced. Both the LST and the in vitro peripheral blood mononuclear cell proliferation assay (CPA) involving soluble *Leishmania* antigen (SLA) are used interchangeably since they have good agreement (98–100%), although the latter is laborious and time-consuming [53]. However, a simpler cellular test currently in use for identifying asymptomatic people in the field is the whole blood stimulation assay and the subsequent detection of cytokines and chemokines in stimulated plasma, which reaches a very high specificity and sensitivity by monitoring the specific expression of IL-2 for *L. infantum* and MIG for *L. donovani* in WBA, while IP-10 production has been described as being useful for both [54]. IL-2 has been already tested in the field [45]. Molecular methods such as PCR have been used less often to identify asymptomatic infection due to undetectable low parasitemia in immunocompetent individuals (0–0.2 parasite/mL blood in asymptomatic individuals compared to 32 to 188,700 parasites/mL in active VL) [55], the difficulty of performing such analyses in the field, and the cost. The new ready-to-use loop-mediated isothermal amplification kit for the accurate diagnosis of leishmaniasis has not yet been tested in the context of asymptomatic carriers. Since the results of using a single test to determine the prevalence of asymptomatic subjects are highly variable and imprecise [55], and in the absence of a gold standard, the combination of several serological/cellular/molecular approaches to accurately estimate the real prevalence of asymptomatic infection is recommended.

Mathematical modelling analysis has proposed that transmission is mainly maintained by asymptomatically infected hosts, on the assumption that although less infective than cases of active VL, their huge number is a significant contributor to transmission [49, 55, 56]. Recent observational studies stress the importance of transmission from clinical cases and post-kala-azar dermal leishmaniasis during epidemic or interepidemic periods, respectively [57]. Until now, the presence of parasites or DNA in the peripheral blood of asymptomatic individuals has not been correlated with positive results by xenodiagnosis [58] (Molina et al., submitted). A recent presentation by S. Sundar at the PKDL meeting held in New Delhi, India (30 July 2019) showed that 183 individuals with high levels of *Leishmania*-specific antibodies were unable to infect sand flies (unpublished results). Therefore, there is not still certainty around the role of asymptomatic carriers in maintaining transmission of leishmaniasis and it needs further evaluation.

On the other hand, asymptomatic *Leishmania* infection has been detected by PCR and LST in HIV+ patients without any history of cutaneous or visceral leishmaniasis [59]. Cellular tests have also been useful for detecting asymptomatic subjects in a cohort of solid organ transplant recipients [60] and of HIV+ patients with no previous leishmaniasis [61].

Years ago, R. Molina et al. confirmed the capacity for infection of HIV-*Leishmania*-coinfected patients by xenodiagnosis [62]. Since then, new highly active antiretroviral therapies (HAART) that help in mounting and/or maintaining a cellular immune response might be contributing to their asymptomatic status, which was not the case in the pre-HAART era. Despite this, there is still a proportion of VL cases in HIV+ patients that need secondary prophylaxis to avoid relapse. In such cases, although they remain asymptomatic for VL, it has been recently demonstrated by xenodiagnosis that they are able to infect sandflies, meaning that this population is still an epidemiological risk (Molina et al., submitted).

In summary, more research is needed to reach a consensus on how to define ‘asymptomatic’ and on the tools to identify carriers as the role they play in transmission may be of paramount importance for elimination/control programmes. Depending on the significance of the asymptomatic population, new chemical entities could be developed as tools for preventive chemotherapy. In addition, research efforts will be required to discover biomarkers and to develop field tests for efficient validation and qualification processes.

#### Filariasis

Filarial nematodes that parasitize humans have developed the ability to modulate the host immune system to assure their long-term survival. Asymptomatic infection is hereby the best trade-off for both organisms, avoiding pathology in the host and ensuring parasite survival. Here we discuss the implications for bystander infections and disease epidemiology.

*Mansonella perstans* infections, for example, are not associated with a specific clinical pathology and are therefore not considered a public health problem [63]. Similarly, although infection with *Loa loa* can lead to extremely high microfilariae loads and adult worms occasionally transit the eye, symptoms are not frequent in endemic populations [64]. Infections with the filarial nematodes *Wuchereria bancrofti*, *Brugia malayi*, *Brugia timori*, or *Onchocerca volvulus*, however, have gained more attention as clinical manifestations may develop. Whereas *W. bancrofti* and *Brugia* spp. cause lymphatic filariasis that can involve lymphoedema of the extremities (elephantiasis) or the scrotum (hydrocele), infection with *O. volvulus* causes onchocerciasis that can result in vision impairment, blindness, or severe dermatitis [65]. However, lymphatic filariasis and onchocerciasis infections do not necessarily cause pathology. Approximately one-third of lymphatic filariasis patients develop oedema in the limb and/or scrotum [66] and 30–50% of onchocerciasis patients develop dermatitis, with ~ 1% of the patients developing its most severe form, also called hyperreactive onchocerciasis or sowda [67].

Clinical symptoms and microfilaremia, the presence of the filarial progeny (microfilariae) that is required for the transmission of the disease, are closely connected to the host’s immune response. As is common for nematode infections, filariae are strong inducers of type 2 immune responses, characterized by eosinophilia, an increased number of innate type 2 lymphocytes, and increased production of type 2 cytokines, and the expansion of IgE antibodies [68]. All of these are important factors for defence against the parasite and are also responsible for the development of clinical symptoms. They are most pronounced, for example, in onchocerciasis patients that develop severe skin dermatitis, which show the strongest Th17 and Th2 immune profiles [67, 69]. Such inflammatory responses are, however, also linked with protective immune responses against the filariae, as sowda patients are often amicrofilaremic [67]. In lymphatic filariasis, around 50% of patients develop microfilaremia. Peripheral blood mononuclear cells (PBMCs) from amicrofilaremic patients have been shown to release more parasite-specific IL-5 and non-specific IL-17 and pro-inflammatory cytokines [70], indicating that increased immune responses are mediating protective immune responses against the filariae; however, this also coincides with the development of pathology. Amicrofilaremic infections are also common for *L. loa* infections [64] and probably also exist for infections with *M. perstans*, although this has not been proven yet.

Filariae can establish an antiinflammatory milieu over time, which leads to the expansion of regulatory cell types such as alternatively activated macrophages or regulatory T cells, increased production of antiinflammatory cytokines, immunosuppressive IgG4, and development of T cell anergy [68, 71]. Regulatory immune responses have evolved to shut down antiparasitic immune responses, facilitating parasite survival, and are associated with asymptomatic filarial infections. Such a suppressive environment can also affect bystander immune responses and therefore alter the outcome of co-infections or immune responses that may otherwise lead to autoimmunity or metabolic disease [68, 72, 73].

Observational studies of co-infections with *Plasmodium* in humans provide evidence that microfilariae-positive infections with filarial nematodes are associated with suppressed *Plasmodium*-specific immune responses and with increased *Plasmodium*-specific regulatory responses [74, 75]. Similarly, *W. bancrofti* and *M. perstans* infections were shown to reduce pro-inflammatory cytokine and chemokine responses during clinical malaria, although clinical signs and symptoms of malaria were not significantly altered, except for increased haemoglobin levels in filariae-infected patients [76]. Similarly, filariasis patients were not associated with changes in the severity of malaria infection.

A similar picture is given for immune responses during latent infections with *Mycobacterium tuberculosis* (MTB), where PBMCs from patients with active lymphatic filariasis had impaired MTB-specific immune responses, but an increased expression of inhibitory molecules [77]. As IFNγ-driven type 1 immune responses are thought to mediate protection against MTB, these results indicate that filarial infection impairs those protective responses during MTB [77]. Nevertheless, results from human studies and animal models are often contraindicatory, as, e.g. co-infections of humans with helminths (including filariae) were not associated with the progression from latent to active MTB or an increased MTB pathology [78]. Indeed, suppression of exacerbated pro-inflammatory immune responses by chronic filarial infection may even provide a beneficial impact, as has been indicated by experimental and human sepsis studies [73].

Furthermore, infections with *W. bancrofti* were identified as a risk factor for acquiring HIV infection [79]. While experimental infections with intestinal helminths have been shown to impair protective immune responses and lead to an increased viral load during co-infection with influenza or murine norovirus [80], a reduction of HIV titres was not observed after antifilarial therapy in *W. bancrofti*–infected patients [81].

The above-mentioned examples demonstrate that filarial infections modulate protective immune responses against other pathogens and may thereby alter clinical pathology.

Current control strategies for onchocerciasis and lymphatic filariasis are based on preventive chemotherapy programmes administered in mapped areas to the at-risk population as a whole (MDA programmes) regardless of whether the individual is infected. Programmes directed by the World Health Organization (WHO) for the treatment and control of filarial disease have been in place for over 40 years and have had a tremendous beneficial impact on public health due to the availability and safety of donated anthelmintic drugs. As MDA targets the population at risk rather than identifying and treating infected individuals, these approaches reach both asymptomatic and symptomatic patients infected with onchocerciasis and lymphatic filariasis.

Limited knowledge about the biology, transmission, clinical aspects of the disease, and impact on public health is currently available for asymptomatic patients infected with filarial nematodes. It has become clear that immunomodulation during asymptomatic filarial infection can impact immune responses during co-infections, but the clinical impact is less well described. Thus, more research is required to understand the impact of filarial infection on the development of MTB and malaria pathology and whether a co-existing filarial infection alters the risk of reactivation of latent MTB or accelerates the development of AIDS. Given that filarial infections also impair immune responses to vaccines [82], it can be hypothesised that asymptomatic filarial infections are of major importance for several infectious diseases and more research is required to pinpoint their exact role. Furthermore, it is well described that filariae produce more offspring in immunosuppressed patients and thus contribute to ongoing transmission.

While amicrofilaremic filariasis patients are not per se asymptomatic and do not contribute to the transmission of filarial disease, there is also a need to investigate these patients in more detail. It is not known whether chronic amicrofilaremic patients can develop microfilaremia later on and thus contribute to the transmission of filarial disease. Since these patients may be missed after the closure of MDA programmes, they present a potential risk factor for re-emerging transmission. Furthermore, diagnostic tools to specifically detect *L. loa* and *M. perstans* adult worm infections in a microfilariae-independent manner are required to identify amicrofilaremic patients, which will allow for the analysis of their contribution to co-infections or as potential reservoir hosts for the transmission of the disease.

#### Malaria

Malaria is a preventable and curable disease, caused by parasites of the *Plasmodium* genus. Across the globe, 3.3 billion people are at risk of malaria, with an estimated 219 million cases and 435,000 deaths in 87 countries in 2017. Global malaria incidence declined by 18% between 2010 and 2017 and was achieved by roll-out of vector control interventions and massive distribution of rapid diagnostics and effective treatment [83]. There has been no further decline in the past 3 years, but rather, the ten highest burden countries in Africa have once again reported increases in the number of cases [83]. Following these initial successes in malaria control, the focus of antimalarial interventions has shifted in many areas from clinical case management to transmission control. Some endemic countries already have achieved, or are aiming to achieve, elimination [84].

Knowledge about carriers of asymptomatic infection is increasingly important for guiding malaria control interventions, particularly in settings of low endemicity, because these individuals mostly remain unnoticed and thus represent an important silent reservoir for onward transmission of *Plasmodium* parasites to the respective *Anopheles* vectors. Improved diagnostic techniques have revealed an unexpected large reservoir of asymptomatic infections. In recent years, convincing evidence on the contribution of asymptomatic infections to onward transmission has become available. Malaria transmission models show that asymptomatic infections, including low-density infections, contribute to ongoing malaria transmission because of the high proportion in the population compared to high density clinical cases [85, 86]. Data on infectivity available so far suggest that light microscopy (LM) and rapid diagnostic tests (RDT) are not sufficiently sensitive to detect the asymptomatic reservoir and that diagnostic methods applied in the field should be adjusted to more sensitive detection for low-density infections [87].

Key public health interventions aimed at asymptomatic parasite reservoirs are drug-mediated strategies, namely (1) mass drug administration (MDA) in an area of transmission irrespective of infection and symptoms; (2) mass screening and treatment with intensive active case detection; (3) intermittent preventive treatment—with repeated treatment of high-risk groups. These approaches have been adopted at different scales and with variable degrees of success, but their combined use is considered an important component of malaria elimination and eradication [88]. Molecular-epidemiological data are very useful for identifying pockets of transmission, particularly in areas of declining malaria transmission, and to guide the choice of control interventions. Molecular tools also provide strategies to validate interventions, but the successful implementation of active case detection is highly dependable on the sensitivity of the diagnostics employed.

Asymptomatic malaria infections and symptomatic clinical episodes have different parasite densities, and thus, the ease of detection is dependent on the diagnostic method for each form. Clinical episodes are generally detectable by blood slide LM or RDT. Differentiation of clinical malaria and non-malaria fevers is complicated by the prevalence of low-density *Plasmodium* sp. infections. Diagnosis of bystander malaria infections may obscure viral or bacterial causes of fever and prevent adequate treatment. RDTs are now widely used as the sole diagnostic for management of clinical malaria. It is safe to withhold antimalarial treatment in infants and young children in all fever cases that are negative by RDT, as has been shown in different endemic settings, including an area with high *P. vivax* prevalence [89, 90]. Such RDT-based treatment strategies have improved the rational use of antimalarial drugs.

Some, but not all, asymptomatic infections can be diagnosed by LM or RDT; however, most malaria infections are missed if no molecular diagnostic test is used. Prevalence rates in the community differ substantially depending on the diagnostics applied. The detection limit of LM and molecular assays differ by several orders of magnitude, i.e. LM and RDT have a detection limit in routine settings of 100–200 parasites/μL blood on average [83], whereas molecular assays can reach a sensitivity as high as 0.03 parasites/μL blood [91], representing an over 1000-fold improvement.

The proportion of submicroscopic *Plasmodium falciparum* infections, i.e. parasitaemia only detectable by PCR, depends on transmission intensity: in regions of low endemicity, the proportion of all *P. falciparum* infections that are submicroscopic may be as high as 80%. Meta-analyses on global data sets compared prevalence rates determined by PCR versus LM and demonstrated that the proportion of submicroscopic *P. falciparum* infections substantially increases with declining malaria transmission intensity [92, 93]. This trend was confirmed in the past few years by numerous molecular-epidemiological studies [94, 95]. Furthermore, a recent study in Zambia, presenting a pre-elimination setting, showed that almost half of all infections remained undetected by RDT [96].

Two meta-analyses that investigated the relationship of *P. vivax* prevalence by LM versus PCR revealed similar trends as found for *P. falciparum* [97, 98]. Both systematic reviews analysed cross-sectional studies from Asia, South America, and the South Pacific with concordant results: high prevalence of submicroscopic *P. vivax* infections and PCR detecting on average 67% more infections than LM. In addition, the proportion of submicroscopic infections was higher in areas of low transmission [97]. Generally, *P. vivax* densities are several-fold lower than those of *P. falciparum* [99]. This represents an additional challenge for *P. vivax* detection. Ultra-sensitive molecular diagnostic assays for *P. vivax* address this limitation by targeting mitochondrial DNA, which is present in a higher number of copies per cell [87, 100].

To increase diagnostic sensitivity for malaria parasites, molecular-epidemiological studies processed large volumes of venous blood or used ultra-sensitive molecular assays that target multiple genomic copies of the molecular marker per cell [101, 102]. A recent study conducted in Papua New Guinea, where *P. falciparum* and *P. vivax* prevalence is equally high, revealed an unexpected high prevalence of asymptomatic sub-microscopic infections, a large proportion (up to 50%) of which was not detected by standard molecular diagnostics (18S rRNA quantitative PCR) using finger-prick blood volumes [91]. This study showed that ultra-low-density malaria infections do not accumulate in certain demographic pockets, thus removing the need to specifically target certain population subgroups if such low-density infections are to be investigated. The diagnostic sensitivity of highly sensitive assays of finger prick blood samples was sufficient for detecting 86% and 91% of the individuals in the community who carry *P. falciparum* and *P. vivax* transmission stages (gametocytes) [91]. Thus, analysis of larger blood volumes revealed a large pool of ultra-low-density *P. falciparum* and *P. vivax* infections, which are unlikely to be transmitted. Additionally, current RDTs, assessed in parallel, cannot replace molecular diagnostics for identifying potential sources of *P. falciparum* transmission.

Solid knowledge now exists on the unexpected high prevalence of asymptomatic infections that are below the microscopic detection threshold and even below the limit of detection of standard molecular techniques. This extensive submicroscopic reservoir has major consequences for malaria surveillance activities, in particular for pre-elimination settings, where most infections are below the detection limit of LM and RDTs and thus would escape diagnosis during malaria control interventions.

Diagnosis of low-density infections currently does not play a role in case management of clinical malaria; however, highly sensitive molecular detection is highly relevant for epidemiological studies, e.g. trials of antimalarial interventions, mapping parasite foci, and determining age-dependent risk factors of infection.

## Discussion and conclusions

The concept ‘asymptomatic’ needs careful definition as it describes a dynamic process. At each stage, the role of the asymptomatic carrier is different. When a human host is infected with a parasite, the host immune response initiates a cascade of events that are specific to the invader and can precipitate three kinds of response: (1) the parasite is eliminated and the infection is overcome; (2) the parasite escapes the host response and invades the tissues, leading to clinically relevant disease preceeded by a premanifest (‘prepatent’) period in which the parasite and its effect(s) on the host are detectable using appropriate methodologies; and (3) the parasite becomes resistant to the host response and enters a chronic phase of disease where both host and parasite co-exist. However, such co-existance is not without consequences, possibly leading to progressive (silent) organ damage and, in addition, may contribute to parasite transmission to new hosts. Although our knowledge of parasitic diseases is considerable, a number of questions pertaining to the asymptomatic period remain unresolved, summarizied in Box 1.

**Box 1:** A number of questions pertaining to the asymptomatic period

The concept of ‘asymptomatics’ is of enormous importance and has become a major topic for research. Frequent questions in the research of asymptomatic carriers are: • Are asymptomatic individuals able to transmit the parasite despite the low burden of circulating parasites? • Is an asymptomatic carrier the same as an asymptomatic individual, as a reservoir for transmission? • Are people carrying a parasite infectious at any moment despite the enhanced immune vigilance of the host, and does this vary across the time of infection? • Can asymptomatic carriers be detected with conventional methods (microscopy, serology, PCR)? • With the current tools, it is possible to distinguish between an asymptomatic carrier and one in the prepatent period? • Among asymptomatic carriers, are there super-spreaders? • Are scientists using the same concepts and terminology when referring to asymptomatic carriers? • Are risk factors that trigger the change from the asymptomatic condition towards clinically impactful disease the same as those that accelerate the prepatent period to florid disease?	

Evidence for the role of asymptomatics as reservoirs for infection and re-emergence and, possibly, new outbreaks is specific to each disease. The role of asymptomatic carriers is probably best described for malaria and Chagas disease, while it is less well understood for lymphatic filariasis, onchocerciais, leishmaniasis, and HAT.

### Chagas disease

Asymptomatic carriers of *T. cruzi* are impacted at both personal and societal levels and affect transmission dynamics. Unlike malaria, the course of Chagas disease in asymptomatic infection is unpredictable, ranging from possible sudden death in the acute phase to a chronic form of disease persisting for decades with few demonstrable clinical symptoms before full manifestation of life-threatening cardiac and digestive disease. The possibility of chronic disease has a psychological impact on patients and family groups and a social impact due to stigmatization and exclusion from potential sources of work. The beneficial impact of antiparasitic treatment for asymptomatic carriers is not widely discussed; those who do not receive treatment have detectable parasitemia cycles, can be a potential reservoir to vectors, are a reservoir for congenital transmission, and act as a potential source for contamination of blood banks when monitoring is not carried out. Early diagnosis and timely antiparasitic treatment are necessary for a favourable impact at individual, social, and epidemiological levels. Diagnosis and treatment of asymptomatic children will result in generations without infection and in women of childbearing age will prevent congenital transmission. The role of asymptomatic individuals in Chagas disease is actively investigated in the research community and the findings applied to disease management.

### Human African trypanosomiasis

Although the existence of human asymptomatic infections with *Trypanosoma brucei* is well documented, their role in transmission remains unknown. Only recently have some research groups, including transmission modellers, prioritized this phenomenon and focused investigations into the underlying mechanism of trypanotolerance, relative frequency of asymptomatics among infected persons, and infectivity potential of trypanotolerant individuals to tsetse flies. It is unlikely that the existence of asymptomatic carriers would compromise the HAT elimination goal, as current strategies targeting patients have successfully led to a drastic decrease of disease prevalence in the last 10 years. Nevertheless, if the role of asymptomatics as a reservoir is confirmed, new strategies will be needed to either sustain elimination or interrupt transmission. In the absence of established prognostic markers for disease progression and in view of HAT elimination, health workers are still confronted with the dilemma of whether or not to treat asymptomatic carriers. This dilemma can only be resolved if new drugs with high benefit/ safety ratios are available for treating otherwise healthy individuals. In the meantime, vector control will continue to be an important tool to control residual transmission of *T. b. gambiense* in endemic populations.

### Leishmaniasis

Most people infected by *Leishmania* species remain asymptomatic and, although they are not thought to play a significant role in transmission, more xenodiagnosis studies are needed to elucidate their role as a reservoir for outbreaks, a frequently observed feature of leishmaniasis. Additionally, biomarkers for identifying the underlying cause(s) for progression from infection to disease would be valuable for epidemiological studies and prevention in endemic areas. Importantly, asymptomatic individuals that become candidates for a programmed immunosuppression for organ transplantation or therapy for autoimmune diseases may require follow-up and surveillance for several years. HIV-positive individuals with asymptomatic infection should be considered potential leishmaniasis cases and therefore suitable for treatment, even though the cellular immune response to the parasite can still be positive. Finally, HIV-positive patients who have been treated for VL and remain asymptomatic but with confirmed capability to infect sandflies have to be considered as ‘spreaders’ and thus should be encouraged to use physical and chemical barriers to avoid being bitten by sandflies. Monitoring the CD4 count and PCR in blood before, during, and after treatment is mandatory.

### Filariasis

Current control strategies for lymphatic filariasis and onchocerciasis use MDA-based approaches, treating the population as a whole, including treatment of patients who are symptomatic, asymptomatic carriers, and persons at risk in endemic areas. However, the contribution of asymptomatic carriers (microfilaremic or amicrofilaremic) to disease transmission is unknown. Following the closure of MDA programmes, amicrofilaremic carriers are likely to be missed, presenting a potential risk for reemergence of disease. Furthermore, the immunomodulation that occurs in asymptomatic filariasis patients impacts co-infections, vaccines, and metabolic and autoimmune responses and therefore presents an often neglected variable in this context.

### Malaria

Asymptomatic individuals carrying malarial parasites have been well characterized, and their role in disease propagation is better understood than for the parasitic infections discussed above. Transmission models show that asymptomatic and low-density infections contribute to transmission, although current diagnostic tests based on light microscopy and rapid diagnostic tests may not be sensitive enough to detect asymptomatic individuals in the field setting, with prevalence rates in the community differing substantially depending on the diagnostic applied. There is a clear need for improved diagnostics to support elimination efforts, as well as more research on the relative cost-effectiveness and operational feasibility of drug-mediated health interventions aimed at asymptomatic parasite reservoirs. Evidence-based guidance on optimum methods for implementing MDA programmes, promoting community engagement and compliance with treatment, and evaluating the effectiveness of MDA programmes is needed. Modelling approaches can inform the optimum method for administering MDA in different epidemiological circumstances and help predict its likely impact.

In malaria and Chagas disease, asymptomatic carriers are clearly acknowledged as a significant risk to control and elimination programs because of their role in transmission and outbreaks. Such clear examples of the role of asymptomatic individuals in evolving disease emphasizes the need for a better understanding of the contribution of asymptomatic individuals in filariasis, HAT, and leishmaniasis, and the possible impact on elimination programs. This is of paramount importance given that HAT in Africa and leishmaniasis in Asia are diseases approaching the elimination target in 2020. In addition, surveillance is required; as in past decades, elimination targets have been nearly achieved only to fall to new outbreaks. More research is needed on asymptomatic individuals to enable the control and elimination of such diseases in the future. DND*i* has focussed on innovating easy-to-use, oral drugs with potential for short duration of therapy to facilitate cure of disease in clinically manifest patients. Hence, it is conceivable that safe, effective therapies can be delivered for treatment of asymptomatic individuals, should they be demonstrated to play a key role in disease transmission or outbreaks. For example, DND*i* has already achieved change in the treatment of HAT with the introduction of NECT in 2009 and the first oral therapy, fexinidazole, in 2019. Should asymptomatic individuals prove to be a reservoir for renewed infections, then acoziborole, a single-dose oral drug with a good safety profile in clinical testing, may have utillity in asymptomatic individuals and populations.

In conclusion, the future is much brighter than at the turn of the century. Drugs intended to be patient-adapted, orally active, and with short treatment regimens are in clinical testing for both leishmanisis and Chagas disease, opening up the possibility of a future where all segments of the patient disease population, both clinically manifest and asymptomatic, can be treated effectively and safely.

## Footnotes

1 In Ancient Greece, offerings and sacrifices to the gods were performed by the priest at an altar, next to it rich and prominent citizens sat around eating the remaining food; they were called ‘parasites’.
